# Computing Integrated Information (Φ) in Discrete Dynamical Systems with Multi-Valued Elements

**DOI:** 10.3390/e23010006

**Published:** 2020-12-22

**Authors:** Juan D. Gomez, William G. P. Mayner, Maggie Beheler-Amass, Giulio Tononi, Larissa Albantakis

**Affiliations:** 1 Department of Psychiatry, Wisconsin Institute for Sleep and Consciousness, University of Wisconsin-Madison, Madison, WI 53719, USA; jgomezvalencia@kettering.edu (J.D.G.); mayner@wisc.edu (W.G.P.M.); mb7989@nyu.edu (M.B.-A.); gtononi@wisc.edu (G.T.); 2 Neuroscience Training Program, University of Wisconsin-Madison, Madison, WI 53719, USA

**Keywords:** causation, regulatory networks, binarization, coarse graining

## Abstract

Integrated information theory (IIT) provides a mathematical framework to characterize the cause-effect structure of a physical system and its amount of integrated information (Φ). An accompanying Python software package (“PyPhi”) was recently introduced to implement this framework for the causal analysis of discrete dynamical systems of binary elements. Here, we present an update to PyPhi that extends its applicability to systems constituted of discrete, but multi-valued elements. This allows us to analyze and compare general causal properties of random networks made up of binary, ternary, quaternary, and mixed nodes. Moreover, we apply the developed tools for causal analysis to a simple non-binary regulatory network model (p53-Mdm2) and discuss commonly used binarization methods in light of their capacity to preserve the causal structure of the original system with multi-valued elements.

## 1. Introduction

Discrete models of biological systems often rely exclusively on binary, or “Boolean” variables with two functional states (“active/inactive”, “present/absent”, or “firing/not firing”). Regulatory networks, in particular, are commonly translated into simplified logical models in order to study the systems’ dynamics and the interactions between network constituents in a qualitative manner [1,2]. Typically, the network elements (for example, genes or proteins) can be idealized as on-off switches around an activity threshold that regulates the levels of expression [3]. However, in some situations, two functional states are insufficient for capturing an element’s behavior adequately, for instance, when an element specifies various effects, depending on different levels of activity [4,5]. This is also the case in neuroscience, where neurons, in their simplest representation, can be viewed as logical elements that either fire or not [6]. Nevertheless, information between neurons (or groups of neurons) may also be conveyed based on different modes of firing, which requires models of neural networks with multiple functional states per element (e.g., 0: low firing, 1: high firing, 2: bursting) [7,8,9,10].

Because the majority of tools and theorems available for the analysis of logical networks are restricted to the Boolean case, binarization methods for converting systems with multi-valued elements into Boolean models have been developed as a way to extend the utility of the available methods [5,11,12,13]. However, these binarization approaches mainly focus on maintaining a model’s asymptotic dynamics, rather than preserving the causal structure of the original non-binary system.

Integrated information theory (IIT) provides a mathematical framework for analyzing the causal structure of discrete dynamical systems [14,15,16]. IIT was originally conceived as a theory of consciousness [17,18,19,20] with the aim of providing a set of requirements that a physical system has to meet to be considered a substrate of subjective experience. To that end, IIT starts by characterizing the essential properties of experience (“axioms”), which are then translated into causal requirements for a system to constitute a physical substrate of consciousness (“postulates”). The main measure, a system’s integrated information (Φ, “Phi”), quantifies the irreducible causal constraints that a system exerts onto itself (above a background of external influences). The more integrated information that a system brings about, the more it can be regarded as a unitary whole as opposed to merely a collection of parts, which makes Φ a measure of causal autonomy [21,22]. Because a system with high Φ needs to be both strongly integrated and differentiated (informative) [23], Φ may also serve as a general measure of complexity [24,25,26]. Consequently, IIT’s mathematical framework has proven to be useful and relevant for research on complexity [24,27,28], emergence [29,30], and certain biological questions [22], in addition to the study of consciousness.

A Python software package (“PyPhi”) that implements the mathematical framework of IIT was released shortly after the latest official update to the IIT formalism (“IIT 3.0”) [14] and it is presented in [31]. PyPhi’s overall functionality has two parts: (1) system-level: to unfold the full cause-effect structure (CES) of a system of interacting elements and to compute its Φ value, and (2) mechanism-level: to identify the intrinsic cause and effect of a particular set of elements within the system and measure its irreducibility (φ, “small phi”).

IIT’s mathematical formalism is generally applicable to discrete dynamical systems with a finite state space. However, so far, PyPhi has been limited to systems of binary elements for reasons of simplicity and efficiency in the implementation and computation. Here, we introduce an extension of PyPhi that makes it possible to compute the cause–effect structures and Φ values of discrete dynamical systems constituted of elements with more than two states. This PyPhi extension to multi-valued elements is now available as part of the original PyPhi package (at: https://github.com/wmayner/pyphi/tree/nonbinary).

In order to demonstrate its utility, in the following we will: (1) analyze and compare random samples of 1000 deterministic and probabilistic systems constituted in varying ways of elements with two to eight states; and, (2) compare the causal properties of a simple non-binary regulatory network model (p53-Mdm2) to its proposed Boolean equivalents. This is followed by a discussion of the utility of binarization methods for understanding the causal structure of a system and the advantages of considering the original system with multi-valued elements. Below, we first provide a brief overview of IIT and the PyPhi functionality. Details regarding the IIT formalism and its implementation for non-binary systems are described in the Methods, Section 5.

## 2. Theory and Pyphi Implementation

Starting from a set of “axioms” that jointly capture the essential properties of phenomenology, IIT proposes a corresponding set of requirements regarding the causal properties of the substrate, which are referred to as “postulates”:

**Existence:** the system must have cause-effect power—it must be able to take and make a difference.
**Intrinsicality:** the system must have cause-effect power upon itself.
**Composition:** the system must be composed of parts that have cause-effect power within the whole.
**Information:** the system’s cause-effect power must be specific.
**Integration:** the system’s cause-effect power must not be reducible to that of its parts.
**Exclusion:** the system must specify a maximum of intrinsic cause-effect power.

Next, IIT uses these postulates as the foundation of a mathematical framework that calculates the cause–effect structure (CES) of any given physical system that is discrete and dynamical, with a finite state space [14,16]. The CES is composed of the set of “causal distinctions” (previously termed “concepts”) [14,32] specified by all mechanisms within the system. A mechanism in IIT is any set of elements within the system that specifies integrated information (φ, “small phi”) about its possible cause and effect within the system by being in its particular current state. Mechanisms that are constituted of a single element are referred to as “first-order” mechanisms, while mechanisms that are constituted of multiple elements are referred to as “higher-order” mechanisms. Note that, by the composition postulate, higher-order mechanisms specify their own cause and effect within the system if they are irreducible to their parts (φ>0) [16]. A mechanism, its cause and effect, and the associated φ value, together, form a causal distinction.

As a measure of a system’s intrinsic cause-effect power, the IIT formalism defines, as its main quantity, the integrated information (Φ, “Phi”) of a system of interacting elements. Φ measures how irreducible a system’s CES is under a system partition that renders part of the system independent from the rest.

Before the experimental results of this manuscript are reported, some relevant terms that are related to the usage of PyPhi need to be introduced. For specifics on how Φ is calculated in the case of multi-valued elements, see Section 5. For information regarding the general usage of PyPhi, we refer to its documentation available at https://pyphi.readthedocs.io and [31].

**Input:** let us begin with the most fundamental item, the Transitional Probability Matrix (TPM). The TPM is a matrix (either deterministic or probabilistic) that specifies the probability with which any state of a system transitions to any other system state, as described in the Methods, Section 5.1. The TPM is determined by the update functions of the system elements and obtained by perturbing the system into all its possible states. It is a matrix of size S×S, where *S* is the total number of possible system states. Moreover, $S=(S_{1}S_{2}\cdots S_{n})$, where $S_{i}$ is the number of states of element *i*.

The TPM is the starting point and—assuming binary elements—is sufficient to compute the integrated information Φ of a system. However, allowing for systems with multi-valued elements requires an additional input that specifies the number of states of each system element (’num_states_per_node’, in our implementation), as systems with different numbers of elements may still have the same total number of states *S* (see below). Moreover, an adjacency matrix may be specified that, as the name indicates, serves to describe who is connected to whom within the network (i.e., a binary matrix). If provided, the adjacency matrix may speed up PyPhi computations. However, because it can also be inferred from the TPM (and the num_states_per_node input), it is not essential to the computation.

**Output:** to obtain the Φ value of a system, as well as its cause-effect structure (CES), which includes the causal distinctions and φ values of the system’s mechanisms, a “system irreducibility analysis” (SIA) is performed. While the full IIT analysis includes a search for the subsystem that specifies a maximum of Φ, our goal here is to compare systems with different types of multi-valued elements. For this reason, we are mainly interested in the properties of the system as a whole. In PyPhi, we thus select the complete set of nodes as the subsystem to be evaluated.

## 3. Results

### 3.1. Comparison of Random Systems with Varying Numbers of Elements and States

Thus far, numerical analyses of the IIT formalism and its quantities has been conducted solely on binary systems (see, for example, [16,24,33]). Extending PyPhi beyond binary systems allows for us to systematically explore the effect of a greater number of states per element on a system’s integrated information (Φ) and cause–effect structure (CES). In general, the capacity for information increases with the size of the state space, and the capacity for composition with the number of system elements. If we hold the number of system elements fixed, having more states per element corresponds to a higher capacity for information due to the larger overall state space. However, if we keep the size of the state space fixed, having more states per element means fewer elements in the system and, thus, fewer possible higher-order mechanisms. This decrease in the capacity for composition may negatively impact the system’s integration.

In order to investigate the interplay between composition, information, and integration in systems constituted of multi-valued elements, we deployed our extended version of PyPhi and analyzed ten different classes of networks with different numbers of elements, topologies, and state spaces, evaluating a set of 1000 networks per class (see Table 1). For comparison, we included two classes of binary systems within the data set. In total, 8000 deterministic TPMs were randomly generated in eight groups of 1000, with sizes: 8 × 8, 9 × 9, two sets of 16 × 16, 27 × 27, 60 × 60, and two sets of 64 × 64.

Because different classes of non-binary systems may have the same total number of states, such classes may share the same TPMs. For example, a two-quaternary-node system with a total of 16 states ($4^{2}$) may have the same TPM as a four-binary-node system with the same total number of states ($2^{4}$). In our data set, networks that belong to class **44(2222)** and **88(444)** required no additional matrices, as they share their TPMs with another class, denoted in parenthesis. Nonetheless, we also included comparison sets with different TPMs for those types of networks (classes **444** and **88**).

**Figure 1:**
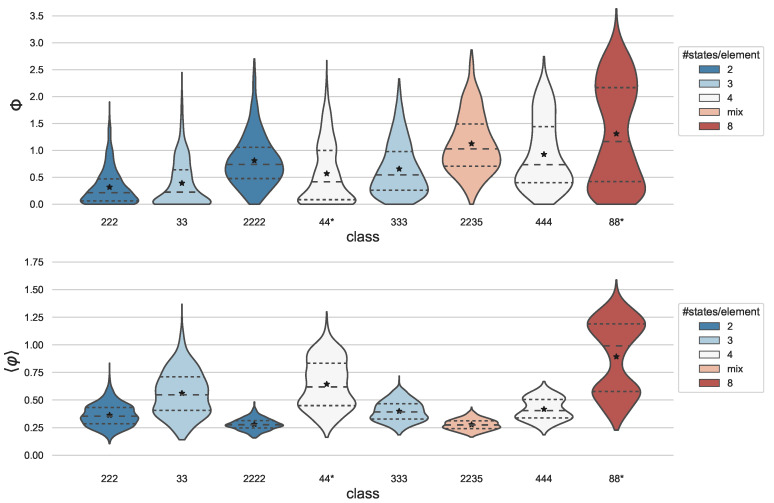
Integrated information across data classes. Plotted are the distributions of Integrated Information (Φ, top) and the average integrated information across a system’s causal distinctions (〈φ〉, bottom) for the various classes in our data set, ordered by the total number of system states from the lowest (leftmost) to the highest (rightmost) (see Table 1). Plots were created with the Python package seaborn, using the violinplot function with parameters cut=0, scale=“count”. Dashed lines indicate quartiles. The mean values are overlaid as stars. Equivalent classes {**44**, **44(2222)**} and {**88**, **88(444)**} were pooled together in this figure (see text). The mean Φ increases with the number of system elements and the number of states per element, while mean 〈φ〉 decreases with the number of elements, but increases with the number of states per element.

For each system in the data set, we computed its integrated information and CES for one state chosen at random from the system’s TPM. In Figure 1, the distribution of Φ, as well as 〈φ〉, the average φ of all causal distinctions within each CES is shown for the different data classes. Here, 〈φ〉 represents a measure of the specificity (information) with which a system’s mechanisms determine their intrinsic causes and effects. We also report the average number of causal distinctions for each class in Table 2, which captures the size of the CES and it corresponds to the number of irreducible mechanisms within the system. By IIT’s composition postulate, a system’s CES may be composed of at most $2^{N}-1$ causal distinctions, where *N* is the number of system elements [16]. These causal distinctions may be specified by “first-order” or “higher-order” mechanisms constituted of one or multiple system elements, respectively. Taken together, the quantities that are reported in Figure 1 and Table 2 reveal complementary aspects of a system’s CES.

As expected, we found no statistical difference between the results of class **44** and **44(2222)**, or between **88** and **88(444)** (a two-sample Kolmogorov–Smirnov test was performed in order to confirm that we cannot reject the hypothesis that the distributions of the two sample pairs are the same; 44*: p=0.65/0.15 and 88*: p=0.91/0.94 for Φ/〈φ〉, respectively). Thus, these classes were pooled together in Figure 1 and Table 2.

As a first variable of interest in our data set, we consider the number of system elements. In line with previous results in binary systems [24], everything else being equal, more elements allow for higher average Φ values (compare **222** to **2222**, **33** to **333**, and **44** to **444**). In contrast, the average 〈φ〉 is lower for the systems with more elements, possibly due to the larger number of available higher-order mechanisms. Even within the same network, higher-order mechanisms typically have lower φ values than first-order mechanisms, because they only specify information to the extent that they are irreducible to their parts (see [14,16]). Indeed, the average number of causal distinctions is already high for class **222** and close to maximal for all other classes in our data set of deterministic random networks (Table 2). When comparing classes with the same number of system elements ({222,333,444}, {33,44,88}, and {2222,2235}), a higher number of states per element resulted in a higher average Φ and also higher average 〈φ〉.

Because Φ depends on the causal interactions between a system’s elements, their composition and integration, the total number of states in the system’s state space is not monotonically related to Φ (compare classes **2222** and **333**, as well as **2235** and **444**). Nevertheless, the total number of system states determines the upper bound of the system’s capacity for information. The TPM of a system, in particular, determines its *effective information* [10,34], which corresponds to the mutual information across a system update from time *t* to t+1 while assuming a uniform distribution of system states at *t*. A system’s effective information is correlated with its intrinsic information (∑φ) for binary random networks, as shown in [16].

**Figure 2:**
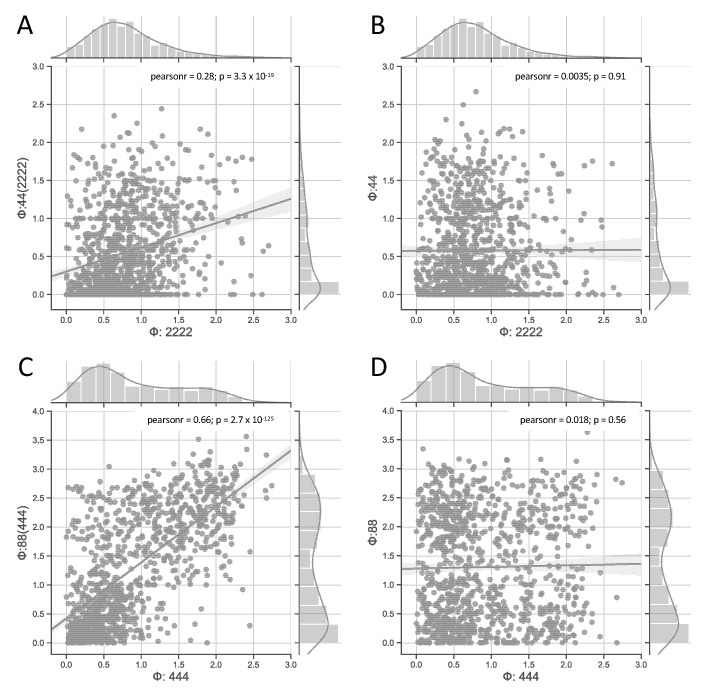
Correlation of Φ values between classes with and without shared TPMs. In this figure, we plotted the relationship between Φ values of several pairs of classes: (**A**) **44(2222)** vs. **2222**; (**B**) **44** vs. **2222**; (**C**) **88(444)** vs. **444**; and, (**D**) **88** vs. **444**. The null correlation between classes **44** vs. **2222** and **88** vs. **444** is expected, as these data samples have different TPMs and are, thus, completely independent. Because the other two pairs share their TPMs, they have the same effective information, but they differ in their causal composition (due to the different number of nodes) and their integration (due to differences in how their nodes are connected). Thus, despite the shared TPM, such network pairs will typically differ in their number of causal distinctions, the corresponding causes, effects, and φ values, and their total amount of integrated information Φ.

In order to explore the role of the TPM across varying numbers of elements and states per element, in Figure 2 we compared those pairs of classes that share their TPMs with the equivalent pairs of classes that have different TPMs. As shown in the figure, both {**2222**, **44(2222)**} and {**444**, **88(444)**} are significantly correlated, while {**2222**, **44**} and {**444**, **88**} are completely unrelated, as expected, since they are independent data samples. Networks with the same underlying TPM necessarily specify the same effective information and the same global dynamics [16]. However, their causal composition and integration depend on the number of interacting elements and their respective number of states. For this reason, the pairs of networks with shared TPMs can be said to have disparate composition and integration (different numbers of elements and different connectivity), but analogous information (TPMs). Thus, the fact that networks of class **444** only have one more element than networks of class **88(444)** may explain why their correlation is stronger than that of {**2222**, **44(2222)**}, which differ by two elements.

### 3.2. Model of Biological Example Systems with Non-Binary Elements

In the following, we will examine the p53-Mdm2 regulatory network model by Abou-Jaoude et al. [5,35], which is discrete, but includes a node with more than two states. We chose this particular example, since discrete network models, as well as the binarization of models with multi-valued elements, are common tools in the study of biological regulatory networks. In particular, we will compare the original model to its Boolean versions that were obtained by means of different binarization methods [11,12,13].

**Figure 3:**
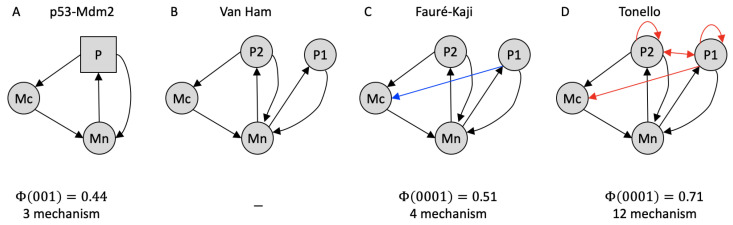
The p53–Mdm2 regulatory network. The arrows indicate causal dependencies, which can be excitatory, inhibitory, or nonlinear. (**A**) Original version with multi-valued element. Circular elements signify Boolean variables, while the square element is multi-valued. (**B**) Binarized version using the Van Ham method [5,11]. Note that this graph is based on an incomplete TPM and thus does not correspond to a complete causal model (see Table 3). (**C**) Under the Fauré-Kaji binarization method [12], **P1** and **P2** become causally equivalent and act jointly on **Mc**. (**D**) The Tonello binarization method [13] introduces additional dependencies between **P1** and **P2**. In all cases, the ternary node P is split into two binary nodes P1 and P2. The Φ values are provided for the fixed point {P,Mc,Mn}=(0,0,1), corresponding to {P1,P2,Mc,Mn}=(0,0,0,1) in the binarized versions. While the Fauré–Kaji method largely maintains the causal structure of the original system, the Tonello method introduces many higher-order mechanisms (see text for details).

The p53-Mdm2 network model describes the interactions between the tumor suppressor protein p53 with its main negative regulator, the ubiquitin ligase Mdm2, with the three variables **P**, **Mn**, and **Mc**, which stand for proteins p53, nuclear Mdm2, and cytoplasmic Mdm2, respectively [35]. **P** takes three values {0,1,2}, while **Mn** and **Mc** are binary variables. In brief, **Mn** down-regulates the level of active **P**, which, in turn, up-regulates the level of **Mc** and also inhibits **Mn**. **P** is modeled as ternary, as it may act on **Mn** and **Mc** above different threshold levels [5,35]. Figure 3A depicts the p53–Mdm2 network as discussed in [5].

For the case of regulatory networks, the “Van Ham” Boolean mapping [11] has been adopted as the standard approach to the problem of transforming multi-level into binary systems. The Van Ham method assigns one Boolean variable to each threshold for each regulatory component in the network. This strategy uniquely preserves neighbor and regulation dynamics under a one-to-one mapping between non-binary and binary system states, as shown in [5]. However, the total state space of the Boolean system is typically much larger than that of the original system with multi-valued elements. Boolean states that do not have a counterpart in the original model with multi-valued elements are considered to be “non-admissible” under Van Ham’s one-to-one mapping [12]. This is problematic, as many tools and results concerning Boolean networks, including IIT’s causal analysis, require fully specified transition probability matrices [12,13,14].

Table 3 lists the evolution function of the p53-Mdm2 network, as specified in [5]. (For causal analysis, we assume synchronous update dynamics identical to the evolution function, which characterizes a system’s interaction graph. In the dynamical analysis of regulatory networks with multi-valued elements, by contrast, it is typically assumed that only stepwise changes of component values are possible [5]—a constraint that is not appropriate for other types of biological models, such as neural networks (see discussion Section 4). In addition, the focus is often put on asynchronous update dynamics that are derived from, but not identical to, the evolution function [12,13].) In the case of the original non-binary system, the evolution function provides the full TPM and, thus, uniquely determines the model’s causal graph (Figure 3A). However, the binary TPM that is inferred by the Van Ham method is incomplete, as it only specifies 12 out of the 16 possible binary states. Only a complete TPM corresponds to a proper mechanistic causal model that specifies how the network components interact. Because the p53-Mdm2 model is deterministic, there are $2^{\left(4\times 4\right)}=65536$ possible ways to populate the four missing rows in the TPM, which may add various causal dependencies to the interaction graph associated with the Van Ham mapping that is shown in Figure 3B.

Recently, two alternative binarization methods have been devised with the problem of non-admissible states in mind [12,13]. Fauré and Kaji [12] provide a binarization method that is applicable to regulatory networks with asymptotic evolution functions, such as the p53–Mdm2 model, in which every component either maintains its current activation level, increases to its maximal value, or decreases to zero. Their method extends Van Ham’s state mapping, such that multi-valued elements are replaced by a set of functionally equivalent binary constituents. This maintains the local interactions between network components, but it leads to a surjective mapping of the dynamical attractors from the binary to the multi-valued implementation. By contrast, Tonello’s method [13] aims to preserve feedback cycles in interaction graphs and a one-to-one mapping of attractors under stepwise, asynchronous update dynamics. To that end, a binary system is created based on a stepwise implementation of the non-binary update function. Using the script that was provided in [12], we computed the binary evolution functions for the p53–Mdm2 network model using the Fauré–Kaji and Tonello mapping, which are presented in Table 3. Figure 3C,D show the causal dependencies between the network components inferred from the respective TPMs.

We performed a full causal analysis for the attractor state {P,Mc,Mn}=(0,0,1), corresponding to {P1,P2,Mc,Mn}=(0,0,0,1) in the binarized versions. Like the original model, the Fauré–Kaji binarization specifies a cause–effect structure that is composed of only first-order mechanisms, while the additional causal dependencies in the Tonello binarization lead to many higher-order mechanisms and, thus, a comparatively higher Φ value. Nevertheless, the Φ value and cause–effect structure of the original system are not identical to those of either binarization.

For asymptotic evolution functions, there exists a causal mapping between the Fauré–Kaji binarization and the original system: the non-binary model can be reconstructed by coarse-graining the binary system obtained with the Fauré-Kaji method as described in [10,29]. In the p53-Mdm2 example, this can be achieved by grouping **P1** and **P2** into the macro element **P** with the following state mapping: {P1,P2}=(0,0)→P=0, {P1,P2}={(1,0),(0,1)}→P=1, and {P1,P2}=(1,1)→P=2. This is not possible with the Tonello method, as **P1** and **P2** have different causal roles within the network.

In order to assess the relationship between the Φ values of systems with multi-valued elements and their Boolean equivalents numerically, we generated three classes of 100 random asymptotic evolution functions (**32**, **43**, and **332**; labels indicate the number of elements and states per element, as described in Table 1). In each case, we evaluated and compared the first state in the system’s TPM. Correlations were consistently stronger between the original system and its Fauré–Kaji Boolean implementation than between the original system and its binarization according to the Tonello method, for which only one condition (**32**) was significantly correlated (p<0.05, Table 4).

While we found a correlation between the Φ values of the original non-binary system and its Fauré–Kaji binarization in all tested samples, the variability is quite large and non-binary systems with Φ=0 may map onto Boolean systems with Φ>0 and vice versa. In order to explore the cause–effect structure and integrated information of a system with multi-valued elements, a causal analysis that is applicable to the actual non-binary system is, thus, essential.

## 4. Discussion

In this article, we have introduced an extension of IIT’s PyPhi toolbox for causal analysis [31] to discrete dynamical systems that are constituted of multi-valued elements. The ability to analyze the causal structure of non-binary systems opens the door to exploring complex networks more representative of those often found in nature. For example, biological regulatory networks are often modeled using multi-valued variables in order to capture dynamics that depend on more than a single activation threshold [4], and the interaction between neurons in a neural network may depend on more than two activity states [7,8,9,10]. Multi-valued causal networks are also commonly investigated in the field of cellular automata [36,37] and multi-valued or fuzzy logic [38,39].

Systems with multi-valued elements may also arise from the coarse-graining of binary networks within the quantitative framework of IIT [10,29] and more generally [40]. Thus, being able to asses the cause–effect structure and integrated information of non-binary networks is relevant for identifying emergent levels of description at which a system’s intrinsic cause–effect power (Φ) reaches a maximum [29].

While our extension of PyPhi to multi-valued elements enables us to move beyond the numerical causal analysis of binary systems, it still comes with the same performance limitations as the original binary implementation [31]: the algorithm is exponential in the number of system elements, which limits the feasible system size to ∼10–12 elements. Moreover, at the moment, the non-binary PyPhi implementation is less efficient than the original implementation (for 100 3-element binary systems: ∼ 32 s vs. ∼ 72 s while using the non-binary code on a MacBook Pro with a 2.4 GHz Quad-Core Intel i5). Note, however, that larger state spaces can be computed for systems with multi-valued elements, as the limiting parameter is the number of elements, rather than the number of states. As before, the analysis can be applied to deterministic and probabilistic systems, but it is limited to Markovian systems that satisfy conditional independence between elements given the past state of the system [14,31].

Everything else being equal, having more states per element increases the size of the state space and, thus, the information capacity of a system [14,29]. As we demonstrated in a numerical analysis of random networks constituted of binary and multi-valued elements in different arrangements, Φ increases with the size of a system’s state space for this type of network; however, not in a strictly monotonic manner (Figure 1). Given the same number of elements, having more states per element, on average, led to higher Φ across all of the tested conditions. In future work, we plan to investigate how the number of states per element affects a system’s integrated information under different types of network topologies, e.g., modular or grid-like architectures.

**Figure 4:**
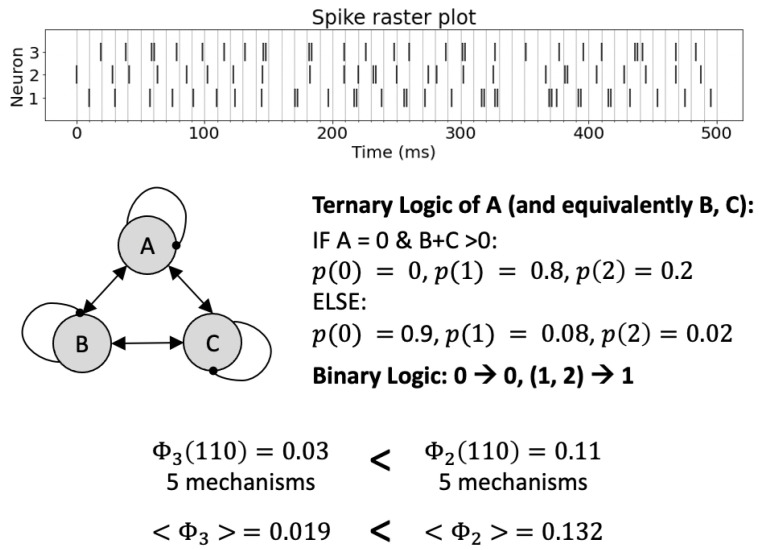
Decreased integrated information in fine-grained system of three interacting neurons. Each neuron fires if it receives excitatory inputs from at least one of the other two neurons and is not currently in a refractory period after firing during the last update (self-loops are thus inhibitory, denoted by round arrow-heads). At each firing, there is a 20% chance that a neuron will emit two action potentials instead of just one (see Ternary Logic). The system’s dynamics are evaluated in 10 ms time bins (top, spike raster plot). Assuming that every neuron has three states (firing 0, 1, or 2 action potentials in one time window) leads to lower values of integrated information when compared to a binary analysis that only distinguishes two states: firing (1) or not (0) when evaluated for the specific state (1,1,0) and also, on average, across all possible states.

While beneficial, it is important to note that having more states per element does not always increase a system’s amount of integrated information. In general, models of biological processes with multi-valued elements are called for in case a system’s functionality requires interactions between its constituents that depend on multiple activation levels [4]. Discretizing, or “fine-graining” a system’s activity levels may lead to a decrease in the system’s intrinsic cause–effect power if the additional states are not, or only marginally, relevant in causal terms (Figure 4, see caption for details). This is relevant, for example, if the TPM is experimentally assessed and the system under study can be sampled at various frequencies and levels of accuracy.

In addition to the information that is specified by the TPM, a system’s integrated information Φ also depends on its composition, which is limited by the number of elements in the system, and their integration (how much information is lost under a partition of the system). Non-binary systems allow us to dissociate the TPM from other network properties, such as the number of elements and their connectivity. For certain sizes of the state space, the same global system dynamics (determined by the system’s TPM) can be implemented with more elements and fewer states per element, or vice versa. While correlated (Figure 2), the intrinsic integrated information (Φ) of such system pairs will typically differ, as the systems are distinct in their composition and integration. How the system elements interact with each other determines the cause–effect structure of the system. Thus, systems with multi-valued elements provide another example that shows that implementation matters for a system’s Φ value (see also [14,16,41]).

As a concrete example for a non-binary biological model, we have applied IIT’s causal analysis to the simple p53–Mdm2 regulatory network and its Boolean translations under different proposed methods for binarization [12,13]. So far, discrete regulatory networks with multi-valued elements have mainly been studied for their dynamical behavior. In that context, binarization methods for facilitating dynamical analysis have been developed with the goal to maintain the asymptotic and, in most cases, asynchronous update dynamics of the system [11,12,13].

IIT provides a framework for studying the mechanistic cause-effect structure and integrated information of discrete dynamical systems, including regulatory networks, which may reveal additional insights about the role of a system’s components and their interactions within the system [22]. Since the evolution function of a system, rather than its asymptotic update dynamics, describes the functionality of the system’s constituents, the evolution function serves as the TPM that is required for IIT’s causal analysis. However, the standard method used for binarization of regulatory networks, Van Ham’s one-to-one state mapping [11] does not provide a unique TPM for the binarized system.

The two subsequently proposed binarization methods that address this issue [12,13] both have particular advantages and domains of applicability (for example, systems with asymptotic [12] or stepwise [13] evolution functions). While these binarization methods were motivated by dynamical rather than causal concerns, we have found that one of them, the Fauré and Kaji method [12], produces a causally “fine-grained” Boolean implementation of the original system for systems with asymptotic evolution functions according to the rules for mapping micro into macro levels of description, as listed in [10]. This is possible if only the average activity of the binary elements that replace a multi-valued element matters for the evolution function of the binarized system. Moreover, based on [29], we conjecture that, for any arbitrary evolution function, a Boolean implementation can be constructed which can be coarse-grained into the original system, if we allow for indeterminism in the binarized evolution function. A ternary element with states {0,1,2}, for example, could then be mapped onto two binary elements, such that both (0,1) and (1,0) equivalently correspond to state (1) of the ternary node. A transition of the ternary node into state (1) would then correspond to a transition of the two binary nodes into (0,1) or (1,0) with equal probability.

While some binarization methods might preserve some aspects of the original cause-effect structure better than others, binarization cannot, in general, provide the same insights as a full characterization of the original non-binary system. Thus, having the tools available to study the causal structure of discrete dynamical systems with multi-valued elements should facilitate the understanding of systems in which the interactions between system elements cannot be characterized in a Boolean manner.

## 5. Methods

**Figure 5:**
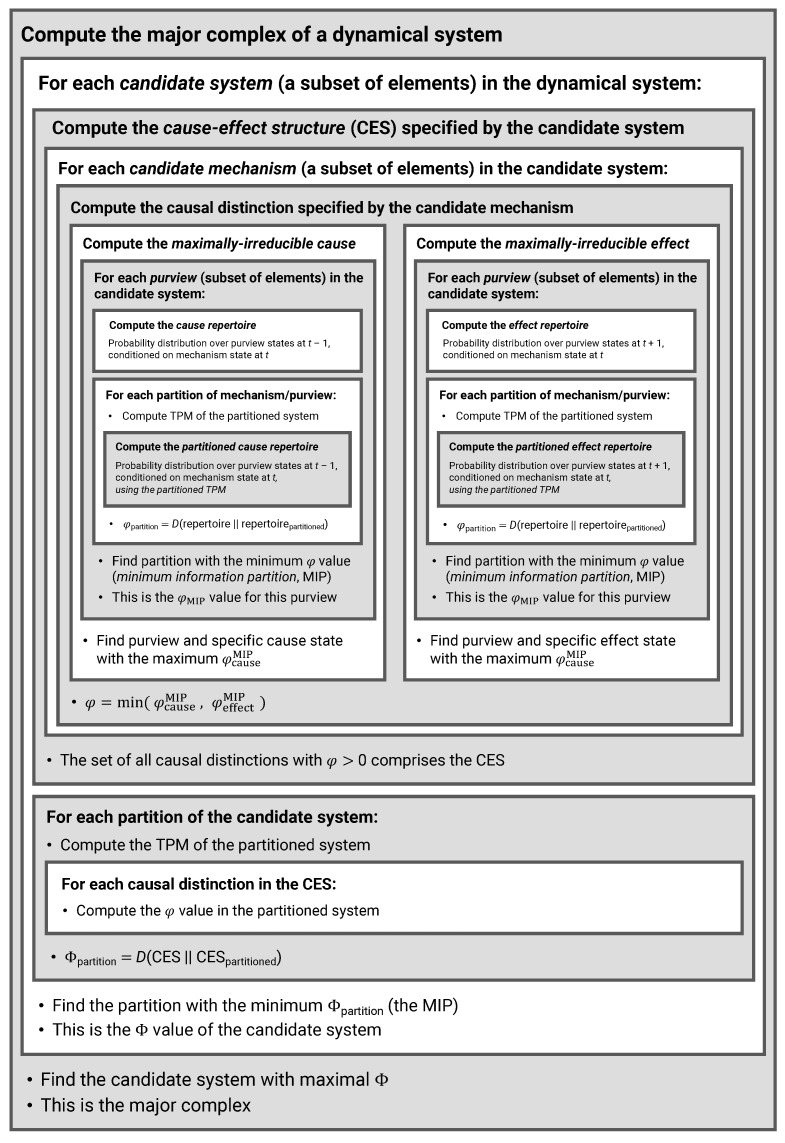
Schematic illustration of the IIT formalism.

The main purpose of this paper is to introduce an extension to PyPhi, a Python package for computing integrated information in discrete dynamical systems with finite state space [31], which allows the evaluation of systems with multi-valued elements. In the following, we highlight differences to the original binary implementation and describe the computation of a system’s CES and its value of integrated information (Φ) in Python-like pseudocode. The extended PyPhi package can be found at https://github.com/wmayner/pyphi/tree/nonbinary. For a mathematical description of the IIT formalism, we refer to [14,15]. Moreover, the original PyPhi publication [31] is accompanied by supplementary material that provides a step-by-step explanation of the IIT formalism. Figure 5 shows a schematic depiction of the formalism.

### 5.1. Non-Binary Implementation

Here, we give an overview of the changes we made to PyPhi in order to implement the calculation for elements with arbitrary numbers of states. First, we discuss some constraints on the form of the TPM for a system of only binary elements that were exploited in the earlier implementation to optimize PyPhi for the binary case (see also [31]); then, we describe how these constraints were relaxed in the generalization of the TPM representation to non-binary systems.

The typical representation of state transition probabilities in a discrete dynamical system with *n* elements is a square matrix P, such that

$$\sum_{j}^{S}P_{i,j}=1,$$

where $P_{i,j}$ is the probability of the transition from state *i* to state *j* and *S* is the number of system states s. This matrix has size $S\times S=\left(S_{1}S_{2}\cdots S_{n}\right)\times \left(S_{1}S_{2}\cdots S_{n}\right)$, where $S_{i}$ is the number of states of element *i*.

In a physical system, causes must precede their effects. Thus, in IIT, it is assumed that the causal model under analysis is sufficiently detailed that there is no instantaneous causation. This assumption is equivalent to the formal requirement that the state $s_{i,t}$ of an element *i* at time *t* is conditionally independent from the current state of all other elements, given the state of its inputs (“parents”) at t−1.

Because $s_{i,t}$ only depends on the state of its parents at t−1, the joint distribution over the states of several elements conditioned on the previous state of their parents can be recovered by multiplication of the conditional state probabilities of individual elements (the Markov property). Thus:

(1) $$\begin{array}{cc} \mathrm{Pr}(s_{t}|s_{t-1})= & \;\mathrm{Pr}\left(s_{1,t}|\mathrm{parents}{\left(s_{1}\right)}_{t-1}\right)\mathrm{Pr}\left(s_{2,t}|\mathrm{parents}{\left(s_{2}\right)}_{t-1}\right)\cdots \mathrm{Pr}\left(s_{n,t}|\mathrm{parents}{\left(s_{n}\right)}_{t-1}\right). \end{array}$$

If the elements of the system each have the same number of states *M*, then the conditional independence assumption of Equation (1) permits a more compact representation of the TPM, termed “state-by-node” form. This is a tensor P with shape S×n×M, where $P_{h,i,j}=\mathrm{Pr}\left(s_{i,t}=j|s_{t-1}=h\right)$. In the binary case M=2, the conditional distributions $\mathrm{Pr}(s_{i,t}=j|s_{t-1}=h)$ have only M−1=1 degrees of freedom, so that the TPM can be represented even more simply as a matrix of size S×n, where $P_{h,i}=\mathrm{Pr}\left(s_{i,t}=1|s_{t-1}=h\right)$. In the binary implementation of PyPhi, this state-by-node form is used as the canonical TPM representation, because it is memory-efficient and allows for the multiplication of distributions to be implemented trivially by taking advantage of NumPy’s broadcasting semantics [31]. However, if elements have varying numbers of states, then the TPM cannot be represented as such a tensor. Thus, for the present work, we represented the TPM using the more typical “state-by-state” form, as an S×S matrix. This required modifying the Network class and implementing new routines for marginalization, which we describe briefly below. For those interested in the details of these changes, the source code is publicly available in the ’nonbinary’ branch of the PyPhi repository (https://github.com/wmayner/pyphi/tree/nonbinary).

We modified the Network class (which represents the dynamical system under analysis) in order to store the number of possible states for each element in the num_states_per_node attribute. This information has to be provided by the user and and it is necessary to keep track of which rows and columns in the TPM correspond to which system states, as the system state is determined by the states of the individual system elements (Equation (1)).

The system’s state-by-state TPM is stored in a Pandas DataFrame, with the rows and columns indexed using a hierarchical MultiIndex. In each index, there is one level per element and the level values correspond to the the element’s states. This allows for indexing into the TPM using state tuples, as in the original implementation (multidimensional state-by-node format).

In order to evaluate a system’s causal distinctions, the IIT algorithm computes cause and effect repertoires for each candidate mechanism (subset of elements) within the system over their possible purviews (again, all subsets of elements) (Figure 5). Cause and effect repertoires are probability distributions over purview states derived from the TPM. To compute the cause or effect repertoire of a mechanism over a purview, nodes not included in the mechanism and purview are marginalized out of the TPM (summing over rows or columns, depending on the case), and the resulting distribution is then conditioned on the state of the mechanism.

Using a Pandas DataFrame allows for the marginalization to be implemented easily with the groupby() method, e.g.,: 

        tpm.groupby(purview, axis= ‘rows’).sum() 

where purview is a list of node labels (i.e., names in the row MultiIndex).

With these modifications, systems of multi-valued elements can be evaluated with PyPhi.

### 5.2. Settings

Because the IIT formalism is currently undergoing several updates (IIT 4.0, forthcoming), some of the changes to IIT 3.0 [14] that have already been included in other publications are also adopted here. In particular, partitions at the mechanism level are defined as in [15,16]; probability distributions are compared using a newly defined “intrinsic difference” measure [42], and Φ is evaluated based on the absolute sum of φ lost from the CES under the system’s minimum information partition (see e.g., [16,43]). To this end, the following PyPhi configuration was used for all computations:

        PARTITION_TYPE = ‘TRI’

        MEASURE = ‘AID’

        USE_SMALL_PHI_DIFFERENCE_FOR_CES_DISTANCE = True

        ASSUME_CUTS_CANNOT_CREATE_NEW_CONCEPTS = True

‘AID’ stands for “absolute intrinsic difference” and implements the intrinsic difference (ID) measure that was introduced in [42]. However, here we evaluate the maximum over the absolute difference between the unpartitioned and partitioned repertoire (see Barbosa et al., forthcoming). In addition to the φ value, the ID also identifies the specific state within the cause and effect repertoire for which the measure is maximal, which corresponds to the specific cause and effect of the mechanism in its current state.

Throughout this study, the above configuration settings apply for both the original PyPhi as well as the non-binary extension. However, note that all of the choices of PyPhi settings relevant for the computation of the CES and Φ are also available for the evaluation of non-binary systems, with the exception of the earth-mover’s distance at the mechanism and system level (MEASURE = ‘EMD’) (see https://pyphi.readthedocs.io/en/latest/configuration.html for the current list of options).

Finally, the IIT formalism also provides the tools for evaluating “relations” between causal distinctions [32]. These will be included in a future PyPhi release and they have not been evaluated for the present study.

### 5.3. Overview of the Algorithm in Pseudocode

The commented Python-like Pseudocode below Algorithm 1 describes the algorithm to identify the major complex (the set of elements with maximal integrated information (Φ)) and all relevant functions to compute the cause-effect structure of a set of system elements in the extended non-binary version of PyPhi. The full source code is publicly available in the ’nonbinary’ branch of the PyPhi repository (https://github.com/wmayner/pyphi/tree/nonbinary).
**Algorithm 1.** Python-like Pseudocode describing the functions used in the extended non-binary PyPhi.
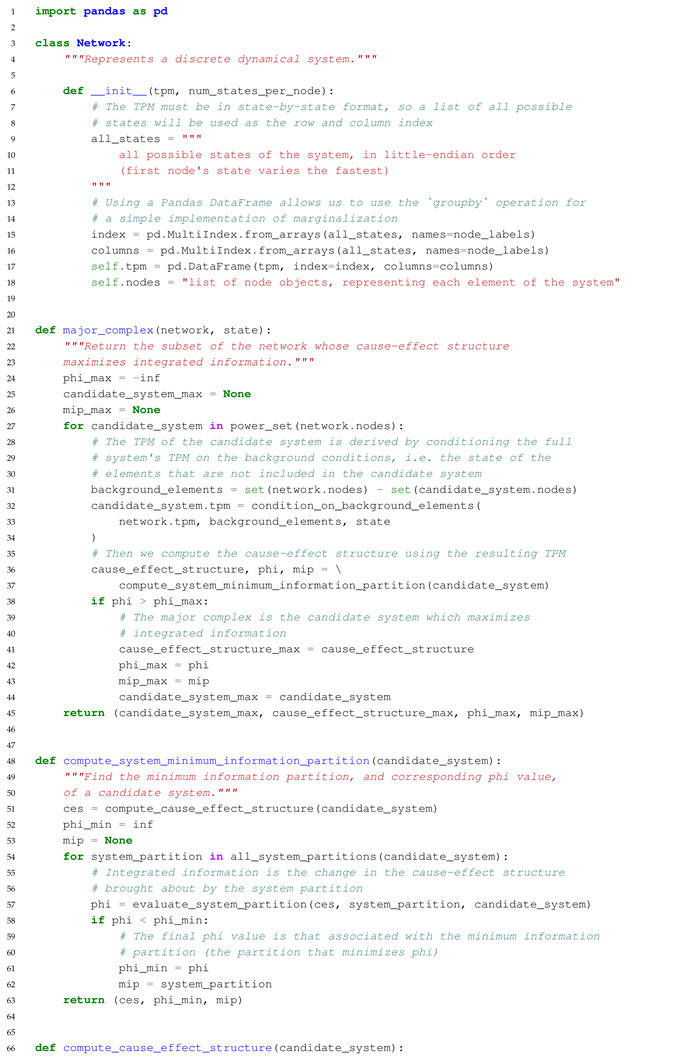

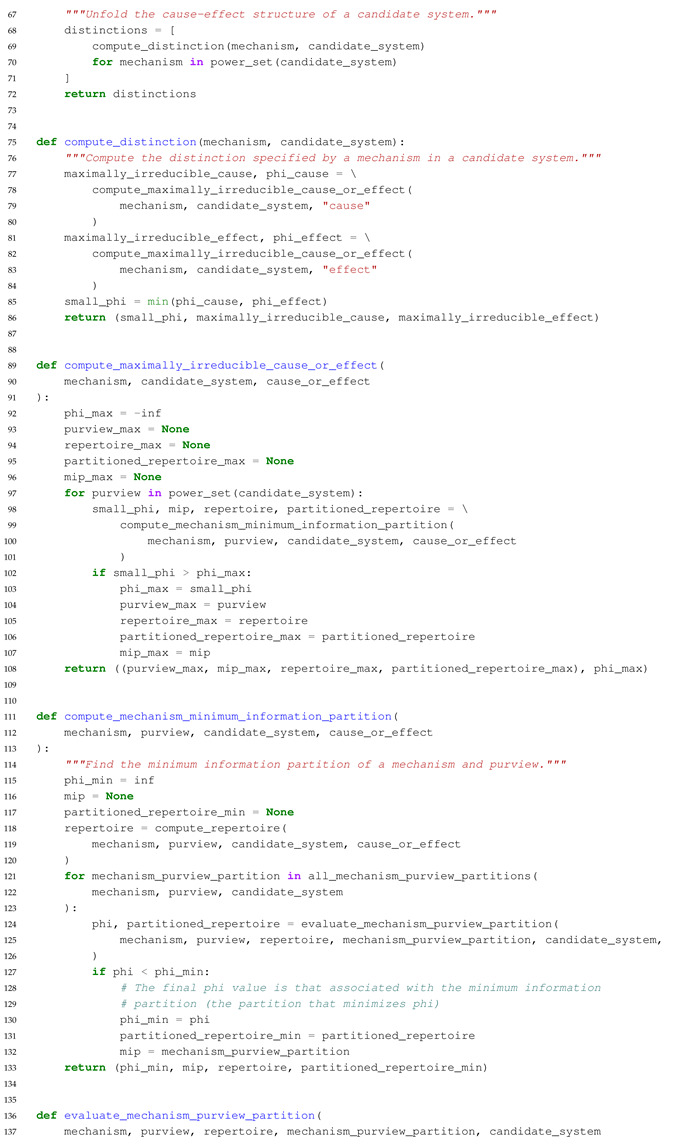

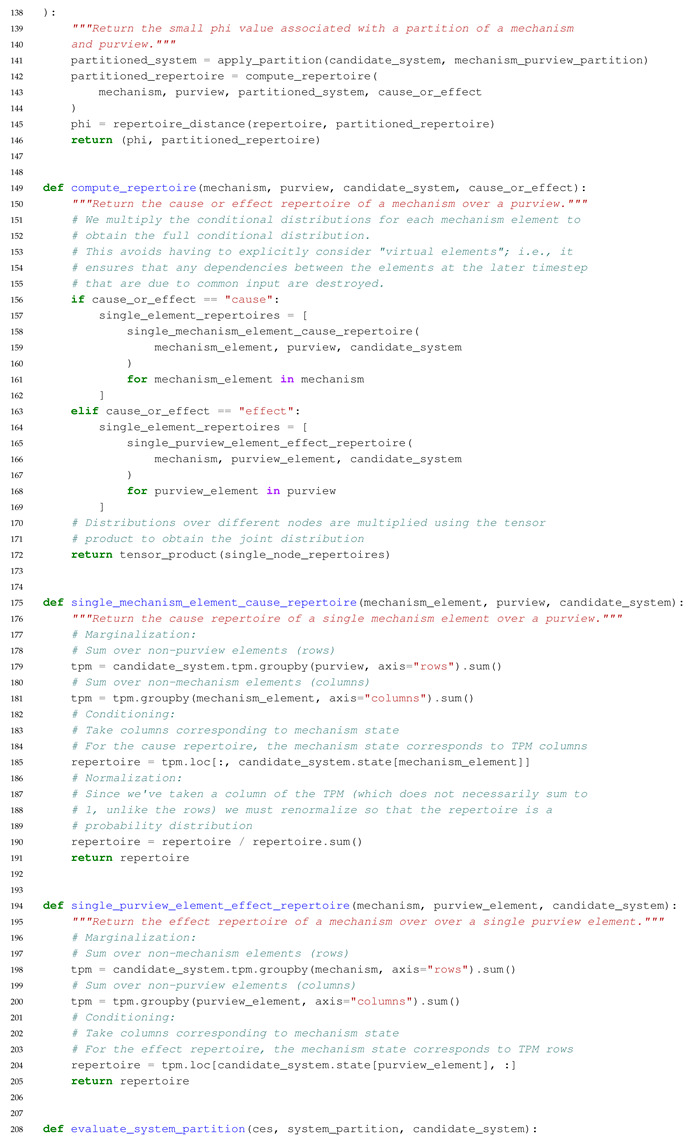

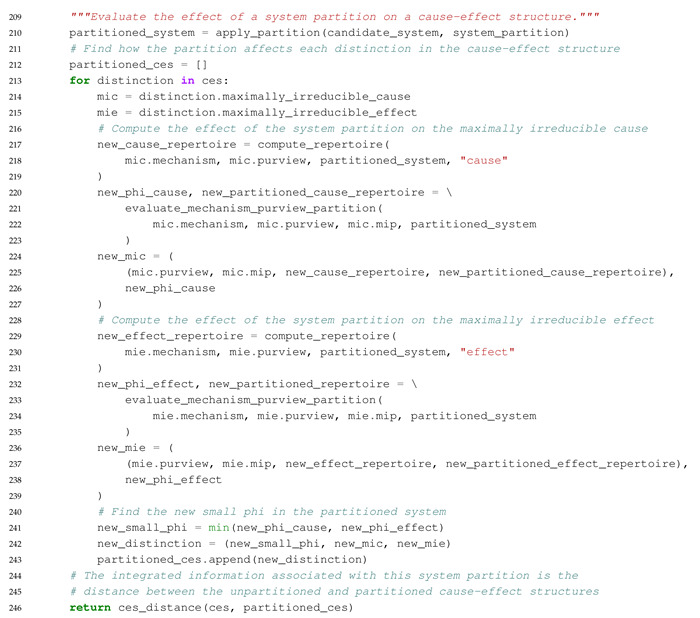



## Figures and Tables

**Table 1:** List of data classes. Classes are labeled such that the number of digits represents the number of nodes of the network, and each digit in turn stands for the number of states of a node. Numbers that are accompanied by parentheses denote that the respective network class shares its TPMs with the one indicated in parentheses. Therefore, **33** corresponds to two-ternary-node networks; **2235** to networks of four nodes with two, two, three, and five states, respectively; and, **88(444)** to two-octal-node networks that share their TPMs with the class of three-quaternary-node networks.

Class	222	33	2222	44(2222)	44	333	2235	444	88(444)	88
**#Nodes**	3	2	4	2	2	3	4	3	2	2
**#States (total)**	8	9	16	16	16	27	60	64	64	64

**Table 2:** Number of causal distinctions per class. The maximum number of distinctions is determined by the number of system elements (*N*) as $2^{N}-1$.

Class	222	33	2222	44*	333	2235	444	88*
(max. #distinctions)	(7)	(3)	(15)	(3)	(7)	(15)	(7)	(3)
**〈#distinctions〉**	5.35	2.71	13.81	2.91	7.	14.95	7.	3.
**% of max**	76%	90%	92%	97%	100%	100%	100%	100%

**Table 3:** Evolution function of p53–Mdm2 network model. The asymptotic evolution function of the original network model with a multi-valued element [5] determines the system’s TPM from state *t* to t+1 (left). The associated Boolean evolution functions generated according to three different binarization methods are shown on the right. The equal sign (“=”) indicates that the state mapping of the Van Ham binarization is maintained for that particular state.

Multi-Valued							Binary															
											Van Ham				Fauré & Kaji				Tonello			
t			t+1				t				t+1				t+1				t+1			
P	Mc	Mn	P	Mc	Mn		P1	P2	Mc	Mn	P1	P2	Mc	Mn	P1	P2	Mc	Mn	P1	P2	Mc	Mn
0	0	0	2	0	1		0	0	0	0	1	1	0	1	=				1	0	0	1
1	0	0	2	0	0		1	0	0	0	1	1	0	0	=				=			
							0	1	0	0	-				1	1	0	0	1	1	0	0
2	0	0	2	1	0		1	1	0	0	1	1	1	0	=				=			
0	1	0	2	0	1		0	0	1	0	1	1	0	1	=				1	0	0	1
1	1	0	2	0	1		1	0	1	0	1	1	0	1	=				=			
							0	1	1	0	-				1	1	0	1	1	1	0	1
2	1	0	2	1	1		1	1	1	0	1	1	1	1	=				=			
0	0	1	0	0	1		0	0	0	1	0	0	0	1	=				=			
1	0	1	0	0	0		1	0	0	1	0	0	0	0	=				=			
							0	1	0	1	-				0	0	0	0	0	0	0	0
2	0	1	0	1	0		1	1	0	1	0	0	1	0	=				1	0	1	0
0	1	1	0	0	1		0	0	1	1	0	0	0	1	=				=			
1	1	1	0	0	1		1	0	1	1	0	0	0	1	=				=			
							0	1	1	1	-				0	0	0	1	0	0	0	1
2	1	1	0	1	1		1	1	1	1	0	0	1	1	=				1	0	1	1

**Table 4:** Pearson correlation coefficients: Φ original system vs. binarizations.

Class		32	43	332
**Fauré-Kaji**	*r*	0.56	0.35	0.29
**method**	*p*-value	≈0	<0.001	<0.005
**Tonello**	*r*	0.24	0.18	0.15
**method**	*p*-value	0.015	0.08	0.14

## Data Availability

All relevant information to reproduce the data analysis presented in this study is contained within the article. The Pyphi software package is freely available at https://github.com/wmayner/pyphi/tree/nonbinary.

## References

[1] Thomas R., D’Ari R. (1990). Biological Feedback.

[2] Abou-Jaoudé W., Traynard P., Monteiro P.T., Saez-Rodriguez J., Helikar T., Thieffry D., Chaouiya C. (2016). Logical Modeling and Dynamical Analysis of Cellular Networks. Front. Genet..

[3] Thomas R. (1973). Boolean formalization of genetic control circuits. J. Theor. Biol..

[4] Thomas R. (1991). Regulatory networks seen as asynchronous automata: A logical description. J. Theor. Biol..

[5] Didier G., Remy E., Chaouiya C. (2011). Mapping multivalued onto Boolean dynamics. J. Theor. Biol..

[6] Dayan P., Abbott L.F. (2000). Theoretical Neuroscience—Computational and Mathematical Modeling of Neural Systems.

[7] Hindmarsh J.L., Rose R.M. (1984). A model of neuronal bursting using three coupled first order differential equations. Proc. R. Soc. London. Ser. Contain. Pap. Biol. Character. R. Soc..

[8] Aizenberg I.N., Naum N., Aizenberg J.V. (2000). Multiple-Valued Threshold Logic and Multi-Valued Neurons. Multi-Valued and Universal Binary Neurons.

[9] Prados D., Kak S. (2006). Non-binary neural networks. Advances in Computing and Control.

[10] Hoel E.P., Albantakis L., Tononi G. (2013). Quantifying causal emergence shows that macro can beat micro. Proc. Nalt. Acad. Sci. USA.

[11] Van Ham P. (1979). How to Deal with Variables with More Than Two Levels.

[12] Fauré A., Kaji S. (2018). A circuit-preserving mapping from multilevel to Boolean dynamics. J. Theor. Biol..

[13] Tonello E. (2019). On the conversion of multivalued to Boolean dynamics. Discret. Appl. Math..

[14] Oizumi M., Albantakis L., Tononi G. (2014). From the Phenomenology to the Mechanisms of Consciousness: Integrated Information Theory 3.0. PLoS Comput. Biol..

[15] Albantakis L., Marshall W., Hoel E., Tononi G. (2019). What caused what? A quantitative account of actual causation using dynamical causal networks. Entropy.

[16] Albantakis L., Tononi G. (2019). Causal Composition: Structural Differences among Dynamically Equivalent Systems. Entropy.

[17] Tononi G. (2004). An information integration theory of consciousness. BMC Neurosci..

[18] Tononi G. (2015). Integrated information theory. Scholarpedia.

[19] Tononi G., Boly M., Massimini M., Koch C. (2016). Integrated information theory: From consciousness to its physical substrate. Nat. Rev. Neurosci..

[20] Albantakis L., Overgaard M., Mogensen J., Kirkeby-Hinrup A. (2020). Integrated information theory. Beyond Neural Correlates of Consciousness.

[21] Albantakis L. (2018). A Tale of Two Animats: What Does It Take to Have Goals?.

[22] Marshall W., Kim H., Walker S.I., Tononi G., Albantakis L. (2017). How causal analysis can reveal autonomy in models of biological systems. Philos. Trans. Ser. Math. Phys. Eng. Sci..

[23] Marshall W., Gomez-Ramirez J., Tononi G. (2016). Integrated Information and State Differentiation. Front. Psychol..

[24] Albantakis L., Tononi G. (2015). The Intrinsic Cause-Effect Power of Discrete Dynamical Systems—From Elementary Cellular Automata to Adapting Animats. Entropy.

[25] Aguilera M. (2019). Scaling Behaviour and Critical Phase Transitions in Integrated Information Theory. Entropy.

[26] Popiel N.J., Khajehabdollahi S., Abeyasinghe P.M., Riganello F., Nichols E.S., Owen A.M., Soddu A. (2020). The Emergence of Integrated Information, Complexity, and ‘Consciousness’ at Criticality. Entropy.

[27] Albantakis L., Hintze A., Koch C., Adami C., Tononi G. (2014). Evolution of Integrated Causal Structures in Animats Exposed to Environments of Increasing Complexity. PLoS Comput. Biol..

[28] Oizumi M., Amari S.i., Yanagawa T., Fujii N., Tsuchiya N. (2016). Measuring Integrated Information from the Decoding Perspective. PLoS Comput. Biol..

[29] Hoel E.P., Albantakis L., Marshall W., Tononi G. (2016). Can the macro beat the micro? Integrated information across spatiotemporal scales. Neurosci. Conscious..

[30] Marshall W., Albantakis L., Tononi G. (2018). Black-boxing and cause-effect power. PLoS Comput. Biol..

[31] Mayner W.G., Marshall W., Albantakis L., Findlay G., Marchman R., Tononi G. (2018). PyPhi: A toolbox for integrated information theory. PLoS Comput. Biol..

[32] Haun A., Tononi G. (2019). Why Does Space Feel the Way it Does? Towards a Principled Account of Spatial Experience. Entropy.

[33] Nilsen A.S., Juel B.E., Marshall W., Storm J.F. (2019). Evaluating Approximations and Heuristic Measures of Integrated Information. Entropy.

[34] Tononi G., Sporns O. (2003). Measuring information integration. BMC Neurosci..

[35] Abou-Jaoudé W., Ouattara D.A., Kaufman M. (2009). From structure to dynamics: Frequency tuning in the p53–Mdm2 network: I. Logical approach. J. Theor. Biol..

[36] Langton C. (1986). Studying artificial life with cellular automata. Phys. Nonlinear Phenom..

[37] Ermentrout G.B., Edelstein-Keshet L. (1993). Cellular automata approaches to biological modeling. J. Theor. Biol..

[38] Gottwald S. (1999). Many-Valued Logic And Fuzzy Set Theory.

[39] Cintula P., Hájek P., Noguera C. (2011). Handbook of Mathematical Fuzzy Logic (in 2 Volumes).

[40] Israeli N., Goldenfeld N. (2006). Coarse-graining of cellular automata, emergence, and the predictability of complex systems. Phys. Rev. E.

[41] Hanson J.R., Walker S.I. (2019). Integrated Information Theory and Isomorphic Feed-Forward Philosophical Zombies. Entropy.

[42] Barbosa L.S., Marshall W., Streipert S., Albantakis L., Tononi G. (2020). A measure for intrinsic information. Sci. Rep..

[43] Krohn S., Ostwald D. (2017). Computing integrated information. Neurosci. Conscious..

